# Totally endoscopic removal of a sewing needle penetrating the left ventricle without cardiopulmonary bypass

**DOI:** 10.1186/s13019-026-03939-8

**Published:** 2026-03-09

**Authors:** Yoshinobu Watabe, Shun Watanabe, Masato Suzuki, Toshiro Ito

**Affiliations:** Department of Cardiovascular Surgery, Sapporo Kojinkai Memorial Hospital, Miyanosawa 2-Jo 1-Chome 16-1, Nishi-Ku, Sapporo, 063-0052 Japan

**Keywords:** Penetrating cardiac injury, Sewing needle cardiac injury, Minimally invasive cardiac surgery

## Abstract

**Background:**

Intracardiac injuries caused by needles are rare and typically require removal via median sternotomy or limited thoracotomy. We present a case of successful removal using a totally endoscopic minimally invasive cardiac surgery (MICS) approach without cardiopulmonary bypass.

**Case presentation:**

A 57-year-old woman presented with chest discomfort one day after falling and striking her left chest. Chest computed tomography revealed a fractured sewing needle, with one fragment embedded in the left ventricular myocardium and the other in the subcutaneous tissue. The patient remained hemodynamically stable, and removal was performed using a totally endoscopic MICS approach was performed. The intracardiac fragment was removed endoscopically without bleeding. The subcutaneous portion was extracted via a small skin incision. The postoperative course was uneventful, and the patient was discharged on day 5.

**Conclusion:**

Totally endoscopic MICS without cardiopulmonary bypass may be a safe and effective option for removing intracardiac foreign bodies in carefully selected patients.

**Supplementary Information:**

The online version contains supplementary material available at 10.1186/s13019-026-03939-8.

## Introduction

Intracardiac injuries caused by foreign bodies such as needles are rare but reported [1–3]. Most cases have required surgical removal via median sternotomy or limited thoracotomy [4]. We present a case in which a fractured sewing needle penetrating the left ventricle (LV) was successfully removed using a totally endoscopic minimally invasive cardiac surgery (MICS) approach [5].

## Case presentation

**Fig. 1:**
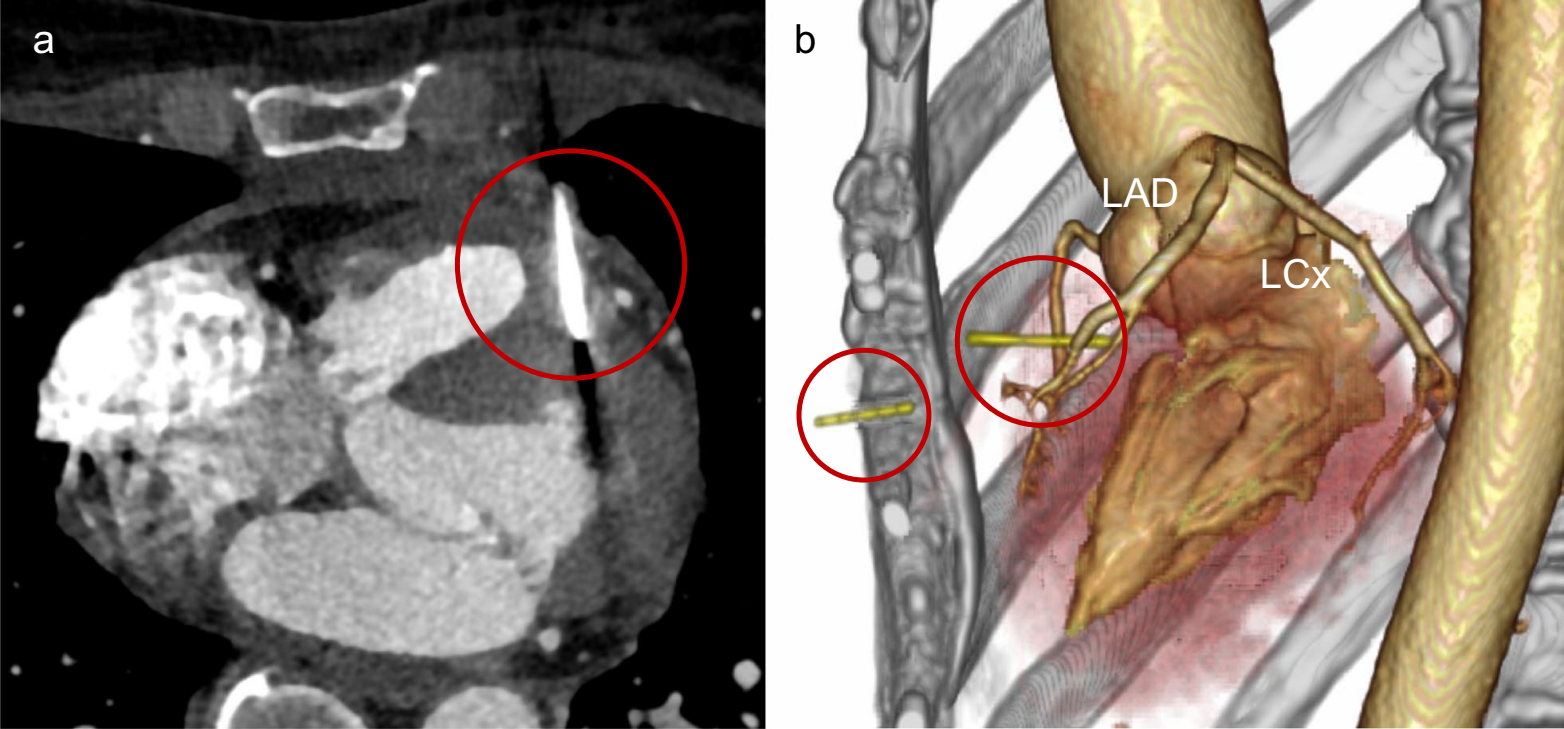
Preoperative CT images. (**a**) Axial CT showing needle fragment embedded in the LV. (red circle). (**b**) 3D-CT showing two needle fragments (red circles) sparing both the left internal thoracic and anterior descending artery. LAD, left anterior descending artery; LCx, left circumflex artery

A 57-year-old woman presented with chest discomfort one day after falling and striking her left chest. Chest computed tomography (CT) revealed a fractured sewing needle, with one fragment embedded in the LV and the other located in the subcutaneous tissue of the left anterior chest (Fig. 1a).

The needle had traversed the LV wall while sparing both the left internal thoracic and the left anterior descending artery (Fig. 1b). Transthoracic echocardiography demonstrated preserved left ventricular function with no evidence of injury to the mitral valve or subvalvular apparatus. As the patient remained hemodynamically stable with no signs of cardiac tamponade, a totally endoscopic MICS approach was selected, and surgery was initiated within a few hours of the diagnostic CT scan.

**Fig. 2:**
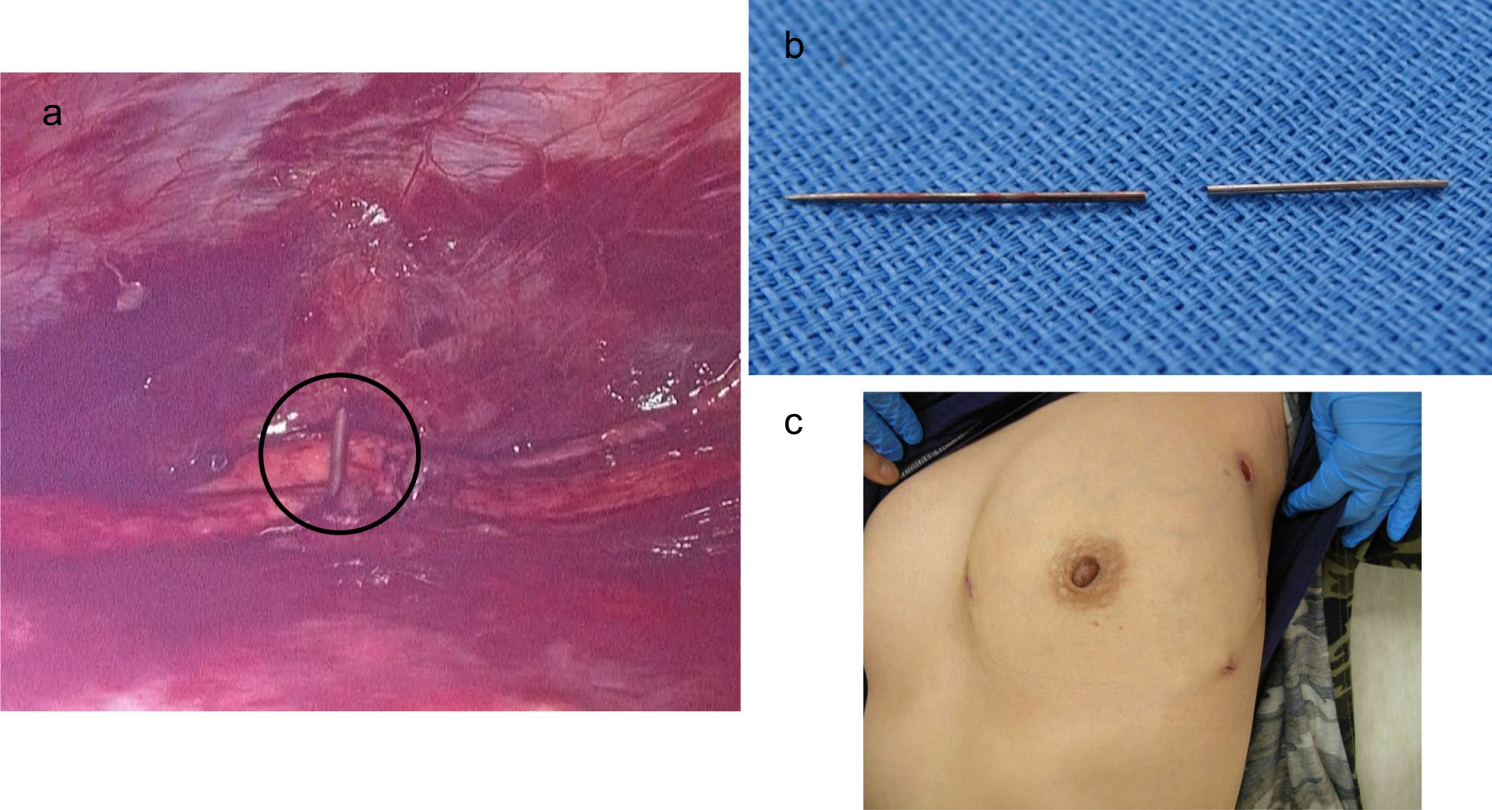
(**a**) Thoracoscopic view of the needle tip penetrating the pericardium. (black circle). (**b**) Extracted needle fragments. (**c**)The cosmetic result after the surgery

The patient was positioned in a 30-degree right lateral decubitus position. A 10-mm 30° oblique-view 3D thoracoscope (Karl Storz, Tuttlingen, Germany) was inserted through the fourth intercostal space on the left anterior-axillary line. Two 5-mm ports were placed at the third and fifth intercostal spaces on the left anterior chest. Hematoma around the anterior pericardial fat was evacuated, exposing the severed end of the sewing needle penetrating the pericardium (Fig. 2a). The intracardiac fragment was carefully extracted with a needle holder under direct thoracoscopic view. The extraction site was observed for approximately 5 min, during which no increase in bleeding was noted and intraoperative transesophageal echocardiography (TEE) confirmed no increase in pericardial effusion. Therefore, no suture repair of the penetration site was performed (Supplementary material Video 1). The subcutaneous fragment, which could not be accessed thoracoscopically, was localized based on preoperative CT findings and skin erythema at the entry site and removed through a small skin incision without fluoroscopic guidance (Fig. 2b, 2c). A 19 Fr Blake drain (Johnson & Johnson, New Brunswick, NJ, USA) was placed through the third intercostal port site. The total operation time was 45 min. Perioperative antibiotic prophylaxis was administered according to institutional protocol, and no postoperative infection, including surgical site infection or infective endocarditis, were observed.

The postoperative course was uneventful. The chest drain was removed on day 2, and the patient was discharged on day 5.

## Discussion

Penetrating cardiac injuries caused by foreign bodies, such as needles have been reported in previous literature. [1–3] In a review by Perrotta et al., 24 cases of needle-induced cardiac injury were analyzed. Most required open surgery via sternotomy or thoracotomy, and none were treated with a thoracoscopic approach [4]. In contrast to these reports, our case demonstrates the feasibility of a totally endoscopic approach without cardiopulmonary bypass in a carefully selected patient.

The anatomical site of injury is a key factor influencing the risk of bleeding and need for surgical repair. In that review, approximately 11 cases involved the LV and 9 involved the right ventricle (RV). LV injuries, subject to higher ventricular pressure, more often required repair, while RV injuries were sometimes managed conservatively. Overall, about 40–50% of cases require myocardial repair. In our case, the needle was embedded within the anterior LV myocardium but did not cause active bleeding or pericardial effusion. This suggests that, in selected cases, thoracoscopic needle extraction from the LV myocardium may be feasible without repair. Also, the small diameter and linear shape of the sewing needle might be key factors supporting simple extraction without myocardial repair. Furthermore, the decision not to suture the penetration site was based on careful intraoperative assessment, including direct thoracoscopic inspection after needle extraction and confirmation by TEE that no pericardial effusion had developed. Importantly, a clear strategy for prompt conversion to open surgery was established in advance in case of postoperative rebleeding or cardiac tamponade.

Several clinical factors supported the MICS approach. First, the patient was stable. Second, preoperative CT confirmed that the needle tip was embedded within the myocardium. Third, the anterior LV wall allowed clear thoracoscopic visualization, and if needed, conversion to an open procedure was considered feasible. The use of a 3D thoracoscopic system further enhanced depth perception and spatial orientation, facilitating precise manipulation and safe extraction of the needle from the left ventricular myocardium. Fourth, the femoral vessels were suitable for emergency extracorporeal circulation. These considerations helped mitigate the risk of hemorrhage and allowed us to proceed safely with a minimally invasive approach. Compared with conventional sternotomy or thoracotomy, a totally endoscopic approach may reduce surgical trauma, postoperative pain, and recovery time in carefully selected patients. Although cardiopulmonary bypass was not routinely established, it was prepared for emergency use, and in the event of active bleeding after needle extraction, immediate compression of the bleeding site followed by prompt conversion to median sternotomy was planned. Based on this experience, hemodynamic stability, intramyocardial localization of the foreign body, favorable thoracoscopic exposure, and readiness for open conversion appear to be key criteria for selecting candidates for a totally endoscopic approach.

Although myocardial repair has typically required open access, direct suturing or pledgeted repair may be feasible under thoracoscopic guidance in accessible areas. This case demonstrates that even LV injuries—typically considered high risk—may be suitable for thoracoscopic treatment in carefully selected patients. Nevertheless, left ventricular pseudoaneurysm remains a potential concern after myocardial penetration, and careful postoperative imaging follow-up is essential. Further accumulation of similar cases will help clarify indications and safety margins for MICS in the setting of penetrating cardiac trauma.

## Conclusions

This case demonstrates that totally endoscopic MICS can be a viable and safe option for the removal of intracardiac foreign bodies in hemodynamically stable patients, offering reduced invasiveness and favorable recovery in selected cases.

## Supplementary Information

Supplementary Material 1: Thoracoscopic evacuation of a pericardial hematoma and removal of a sewing needle penetrating the left ventricular myocardium. No bleeding or increase in pericardial effusion was observed after extraction.

## Data Availability

All data generated or analyzed during this study are included in this published article and its supplementary materials.

## Abbreviations

MICS: Minimally invasive cardiac surgery
CT: Computed tomography
LV: Left ventricle
RV: Right ventricle
TEE: Transesophageal echocardiography
